# Isolation and Chemical Characterization of a Toxin Isolated from the Venom of the Sea Snake, *Hydrophis torquatus aagardi*

**DOI:** 10.3390/toxins1020162

**Published:** 2009-12-08

**Authors:** Masaya Nagamizu, Yumiko Komori, Kei-ichi Uchiya, Toshiaki Nikai, Anthony T. Tu

**Affiliations:** 1 Department of Microbiology, Faculty of Pharmacy, Meijo University, Nagoya 468-8503, Japan; Email: ykomori@ccmfs.meijo-u.ac.jp (Y.K.); kuchiya@ccmfs.meijo-u.ac.jp (K.U.); nikai@ccmfs.meijo-u.ac.jp (T.N.); 2 Department of Biochemistry and Molecular Biology, Colorado State University, Ft. Collins, CO 80523, USA

**Keywords:** Hydrophiidae toxin, *Hydrophis torquatus aagardi* toxin, sea snake toxin

## Abstract

Sea snakes (family: Hydrophiidae) are serpents found in the coastal areas of the Indian and Pacific Oceans. There are two subfamilies in Hydrophiidae: Hydrophiinae and Laticaudinae. A toxin, aagardi toxin, was isolated from the venom of the Hydrophiinae snake, *Hydrophis torquatus aagardi* and its chemical properties such as molecular weight, isoelectric point, importance of disulfide bonds, lack of enzymatic activity and amino acid sequence were determined. The amino acid sequence indicated a close relationship to the primary structure of other Hydrophiinae toxins and a significant difference from Laticaudinae toxins, confirming that primary toxin structure is closely related to sea snake phylogenecity.

## 1. Introduction

There are many varieties of venomous snakes in the world. Among them, sea snakes are unique in that they spend most of their life in the sea. The family of sea snakes, Hydrophiidae, is divided into two subfamilies: Hydrophiinae and Laticaudinae. Sea snake venom is a mixture of proteins with diverse biological activities [1,2]. In our laboratory, we have isolated and characterized neurotoxins from many species of Hydrophiinae sea snakes [3,4,5,6]. In this report, we present data on the isolation of a toxin from the venom of *Hydrophis torquatus aagardi* belonging to the Hydrophiinae subfamily. We compared the various chemical properties of this toxin we named “aagardi toxin” with other Hydrophiinae toxins and also with other land snake toxins. Since the chemical structure is related to the phylogenicity of sea snakes, it is valuable to compare these properties of different toxins.

The structure-function relationship of a neurotoxin is very important. However, the first step towards reaching this goal is to establish the exact primary structure first. As a result, in this paper we emphasized the primary structure, though we would like to pursue more functional aspects of the toxin in the future.

## 2. Materials and Methods

Specimens of the sea snake, *Hydrophis torquatus aagardi*, were captured in the Gulf of Thailand. The venom glands were removed and dried at room temperature. Crude venom was extracted from ground venom glands with distilled water and the insoluble tissue debris was removed by centrifugation (3,860 g, 30 min, 4 °C), then the supernatant liquid was lyophilized for storage.

### 2.1. Isolation procedure

All of the purification procedures were performed at 4 °C. After each purification step, all pooled fractions were tested for toxicity. Crude venom (10 mg) was dissolved in 5 mL 10 mM potassium phosphate buffer (pH 7.8) and fractionated using a Sephadex G-50 column. The toxin was eluted with the same buffer at a flow rate of 13.5 mL per hour. The eluate, collected in 3-mL aliquots, was monitored at 280 nm. The fractions which possessed lethal activity were pooled and applied to a CM52-cellulose column. The column was eluted with a stepwise elution of 0.05 M and 0.1 M NaCl in 10 mM potassium phosphate buffer (pH 7.8) at a flow rate of 13.5 mL per hour. The eluate, collected in 3-mL aliquots, was monitored at 280 nm.

### 2.2. Toxicity test

The toxicity tests were done by injecting 0.1 mL (in saline) of toxin at various concentrations intravenously into ddY mice weighing 20 g each. At each of the five dosage levels, eight mice were used. The number of mice died within 24 hours was observed. The toxicity was determined statistically using the method of Litchfield and Wilcoxon [7] and expressed as the lethal dosage 50%, the LD_50_ value (micrograms of toxin per gram of body weight of mouse). Experimental protocols concerning the use of laboratory animals were approved by the committee of Meijo University.

### 2.3. Homogeneity

The homogeneity of the toxin was checked using SDS-polyacrylamide gel electrophoresis (SDS-PAGE). Electrophoresis was performed on SDS-PAGE using MULTIGEL II Mini 15/25 (13W). The sample was treated with 0.5 M phosphate buffer (pH 7.2) containing 2% SDS and reduced with 3% b-mercaptoethanol at 100 °C for 3 minutes before electrophoresis on polyacrylamide gels. SDS-PAGE was carried out on 12% polyacrylamide gels using a constant current of 12 mA at 25 °C for 2 hours.

### 2.4. Enzymatic cleavage

The purified toxin (500 mg) was digested with trypsin (25 mg) in 1 mL of 100 mM Tris-HCl buffer (pH 7.8) at 37 °C for 2 hours. The purified toxin (500 mg) was digested with endoproteinase Arg-C (5 mg) for 2 hours at 37 °C in 1 mL of 100 mM Tris-HCl buffer (pH 7.8) containing 10 mM CaCl_2_. All digests were separated by reversed-phase HPLC. 

### 2.5. Primary structure analysis

The amino-terminal sequence of native toxin and the enzymatically cleaved fragments of toxin were analyzed by an Applied Biosystems 491 protein sequencer. The phenylthiohydantoin (PTH) derivatives of amino acids were identified with an Applied Biosystems Model 610A PTH analyzer in accordance with the manufacturer’s instructions.

### 2.6. Molecular mass

The molecular mass of purified toxin was determined by MALDI/TOF-MS (matrix-assisted laser desorption/ionization time-of-flight mass spectrometry) with a Nihon Perseptive Biosystems/Voyager R.P., and by SDS-PAGE using the method of Weber and Osborn [8]. SDS-PAGE was performed by using phosphorylase b (94,000 Da), bovine serum albumin (67,000 Da), ovalbumin (43,000 Da), carbonic anhydrase (30,000 Da), soybean trypsin inhibitor (20,100 Da) and lactalbumin (14,400 Da) as the protein standards.

### 2.7. Isoelectric focusing/polyacrylamide gel electrophoresis

The Pharmalyte concentration used for isoelectric focusing was 3% (w/v) with a pH range of 3-10. Acetylated cytochrome C, pI 10.6, 9.7, 8.3, 6.4, 4.9, and 4.1 were used as the protein standards. Isoelectric foucusing was carried out using a constant potential of 200 V at 5 °C for 4 hours.

### 2.8. Other activities

Various enzymatic and biological activities were determined by following published procedures: Phospholipase A_2_[9], arylamidase [10], proteinase [11], arginine ester hydrolase [12], fibrinogenolytic enzyme [13], elastase [14], and hemorrhagic activities [15].

## 3. Results

### 3.1. Isolation and purification

**Figure 1:**
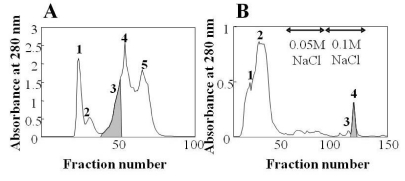
Elution profiles for the isolation of aagardi toxin from *H. t. aagardi* venom. (A) The first step: *H. t. aagardi* crude venom (10 mg) was applied to a column of Sephadex G-50 (1.5 × 100 cm) equilibrated with 0.01 M potassium phosphate buffer (pH 7.8). The column was eluted with 0.01 M potassium phosphate buffer (pH 7.8). Fractions of 3.0 mL were collected at a flow rate of 13.5 mL/hr. (B) The second step: Peak 3 from the first step was applied to a column of CM52-cellulose (1.5 × 45 cm) equilibrated with 0.01 M potassium phosphate buffer (pH 7.8). The column was eluted with a step gradient of 0.01 M potassium phosphate buffer (pH 7.8) containing 0.05 M and 0.1 M NaCl. Fractions of 3.0 mL were collected at a flow rate of 13.5 mL/hr.

Aagardi toxin was isolated by a combination of gel filtration and CM52-cellulose column chromatographies. Lethal activity was found in the fractions indicated by shading (Figure 1A). This fraction 3 was further purified by using anion exchange column, and the lethal fraction 4 gave a single band establishing the homogeneity of the preparation. This purified toxin was named aagardi toxin (Figure 1B).

**Figure 2:**
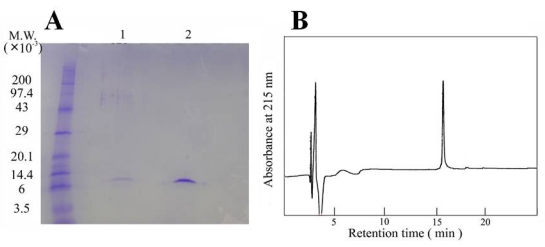
Homogeneity of aagardi toxin. (A) SDS-PAGE of aagardi toxin under reduced (lane 1) and non-reduced (lane 2) conditions. (B) Fractionation by reversed-phase HPLC of aagardi toxin. Column: Develosil 300 ODS-HG-5 (4.6 × 250 mm). Solvent A: 0.1% TFA in H_2_O, Solvent B: 0.1% TFA in acetonitrile. Flow rate 1.0 mL/min. Elution achieved over 40 minutes with a linear gradient from 0 to 60% using solvent B.

Homogeneity of the toxin was established by two independent methods: SDS-PAGE (Figure 2A) and reversed-phase HPLC (Figure 2B). In each case, a single band was observed. The yield of the aagardi toxin from 10 mg of *H. torquatus aagardi* venom was found to be 1.9 mg. 

### 3.2. Toxicity

The LD_50_ of the aagardi toxin in mice was found to be 0.036 (0.019~0.068) mg/g by intravenous injection, indicating the extremely toxic nature of the toxin. The LD_50_ of purified sea snake neurotoxin from *Pelamis platurus* by *i.v.* was 0.13 mg/g [4]. A purified toxin from *Hydrophis ornatus* gave an LD_50_ value of 0.09 mg/g by intramuscular injection [16]. Therefore, the toxicity of *H. torquatus aagardi* toxin is high compared to the neurotoxins isolated from *P. platurus* and *H. ornatus*. The effect of temperature on toxicity was also examined. The results of injection of 1.5 mg toxin that had been heated for ten minutes. At each of the three temperature levels, three mice were used.

The result shows the high thermal stability of the toxin. At 70 °C, all mice in the group died, which meant that the toxin retained full potency. Even at 85 °C, two out of three mice in the group died, indicating the toxin is still active at high temperatures. The toxin was denatured in the test at 100 °C, as indicated by the survival of all three test animals. 

### 3.3. Chemical properties

The molecular mass of aagardi toxin was determined by SDS-PAGE to be 7,400, while the MALDI-TOF/MS method gave a molecular weight of 6,591.10. The isoelectric point was determined by electrophoresis and was found to be higher than 10.0.

### 3.4. Reduction of disulfide bonds

It is known that most sea snake toxins contain four disulfide bonds, and only a few of them contain five. Disulfide bonds are especially important for small proteins in order to hold the peptide backbone together. The reagent dithiothreitol (DTT) is known to cleave disulfides by reducing the bond. When 100 mL of aagardi toxin at a concentration of 15 mg/mL was incubated with 1% DTT at 37 °C and then injected into mice intraperitonially, all three mice survived. This indicates that the disulfide bonds are essential for toxicity because the cleavage of these bonds with the reducing agent resulted in a loss of toxicity. When neurotoxin is reduced chemically, all four disulfide bonds are broken. Therefore, at this stage it is not possible to pin down exactly whether one, two, or three disulfide bonds are essential.

### 3.5. Enzymatic and biological assays

In order to ascertain that aagardi toxin is not an enzyme and does not show hemorrhagic and clotting activity, some assays were performed. The following assays yielded negative results: Phospholipase A_2_, elastase activity using Suc-Ala-Ala-Ala-pNA as the substrate, arginine ester hydrolase activity using TAME (tosyl-L-arginine methyl ester) and BAA (Bz-L-arginine- NH_2_·HCl·H2O) as substrates, arylamidase activity using L-leucine b-naphtylamide as the substrate, proteinase activity using dimethylcaesin, fibrinogen, bradykinin, casein, and insulin B chain as substrates, collagenase activity using collagen Type IV as the substrate, blood clotting activity, and hemorrhagic activity.

### 3.6. Fragments

**Figure 3:**
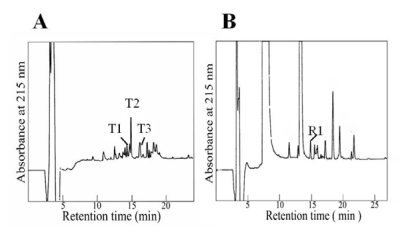
Fractionation by reversed-phase HPLC of peptides obtained by digestion of aagardi toxin with trypsin (A) and endoproteinase Arg-C (B). Elution conditions were the same as described in Figure 2B.

**Figure 4:**
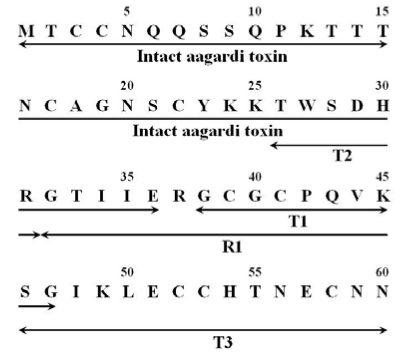
Amino acid sequence of aagardi toxin. Arrows indicate residues determined by sequence analysis. The following abbreviations are used for the some peptides: T, trypsin; R, endoproteinase Arg-C.

Aagardi toxin was digested with trypsin and different fragments were isolated by reversed-phase HPLC as shown in Figure 3A. The amino acid sequence of each fragment (T1, T2, and T3) was determined as shown in Figure 4. By endoproteinase Arg-C digestion, the fragment R1 was isolated and its amino acid sequence was determined (Figure 3B). 

The purified toxin was subjected to direct amino acid sequence analysis and the sequence up to residue 36 was established (Figure 4). By combining all the data obtained from the whole toxin and various fragments, the complete amino acid sequence of aagardi toxin was determined (Figure 4), and aagardi toxin is composed 60 amino acids and the molecular mass of the protein portion of aagardi toxin was examined to be 6,591.19 Da. The molecular mass of aagardi toxin based on the amino acid sequence was identical to the molecular mass (6,591.10 Da) obtained by MALDI/TOF/MS.

## 4. Discussion

### 4.1. Stability of sea snake toxins

It is well known that sea snake toxins are relatively stable to heat because of their compact molecular structure with a relatively small size and a peptide backbone held together by four disulfide bonds [1]. The present investigation shows that aagardi toxin is no exception, showing stability at the relatively high temperature of 85 °C. The stability of sea snake toxins is derives from the disulfide bonds in holding the molecule together, explaining why toxicity is lost when the disulfide bonds are reduced with DTT.

### 4.2. Non-enzymatic nature of sea snake toxins

As compared to the venoms of land snakes, the enzymatic activity of sea snake venoms has rarely been studied. Presynaptic toxins are known to have phospholipase A_2_ activity; it is for this reason that it is important to investigate the enzymatic activity of aagardi toxin.

A variety of enzyme tests yielded negative results. Blood clotting activity that is also due to the activity of many proteolytic enzymes was shown to be negative as well. Finally, hemorrhagic activity, also due to a different proteolytic enzyme, was shown to be negative as well.

These results amply prove that this sea snake neurotoxin is a non-enzymatic type toxin. This is consistent with earlier findings that sea snake toxins bind to the acetylcholine receptor and are competitive inhibitors of acetylcholine [6,17].

### 4.3. Comparison of primary structure within Hydrophiinae toxins

**Figure 5:**
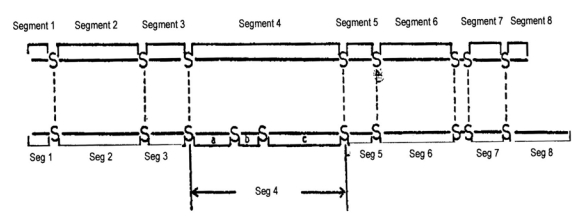
Schematic diagram of short and long neurotoxins showing similarities in the relative positions of half-cystine residues.

In order to compare different toxins better, the toxin is broken down into eight segments. These segments are shown in Figure 5, which was originally published by the Annual Review of Biochemistry in 1973 [1].

**Figure 6:**
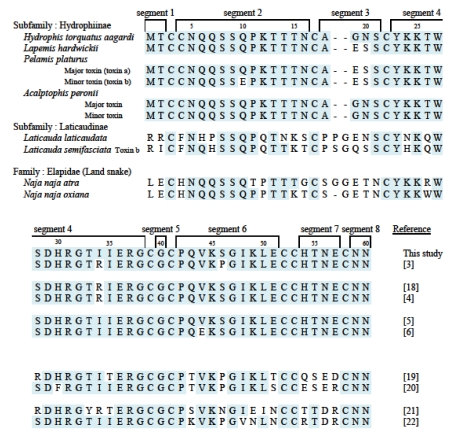
Comparison of the amino acid sequence of aagardi toxin, some sea snake and Elapidae snake neurotoxins.

By comparing sequences of Hydrophiinae toxins, it becomes apparent that the disulfide bonds are conserved because of the presence of cysteine residues at the same places. The remaining residues are also extremely similar among the toxins (Figure 6). The differences are few and are summarized here.

1. Position 10: Most toxins show Q (glutamine), with only *Pelamis platurus* minor toxin having E (glutamic acid).
2. By comparing the amino acid residues it is apparent that the amino acid residue at 19 is E for Pelamis minor toxin and G for aagardi toxin (Figure 6). At position 34, R for Pelamis major toxin becomes I for aagardi toxin. It is premature to conclude definitely, however the differences at 19 and 34 may be correlated with toxicity difference.
3. As compared to the venoms of land snakes, enzymatic activity of sea snake venoms has rarely been studied. Therefore, we really don’t know the enzyme activity until we actually test. Presynaptic toxins are known to have phospholipase A_2_ activity; it is for this reason that it’s worthwhile to investigate such activities.
4. Position 44: Most toxins have V (valine), and only one toxin shows E.
5. Position 46: Most are V; only *Lapemis hardwickii* shows P (proline).

### 4.4. Comparison of Hydrophiinae and Laticaudinae toxins

Only two toxins from subfamily Laticaudinae are quoted here for comparison. There are still many similarities, especially the disulfide bond backbone, but there are clearly more differences between the two subfamilies when analyzing the amino acid sequence of the neurotoxins than in an analysis of the sequences of toxins within the Hydrophiinae.

### 4.5. Comparison with land snake (Elapidae) toxins

Two cobra toxins are presented here for comparison. Elapidae toxins are quite different from all Hydrophiinae toxins, especially in segments 3, 6, and 7. Comparison of the toxin sequences shown here reveals that primary structure is related to the phylogenicity of snakes: the more closely related, the more similar the amino acid sequence.
